# Supplementary figures and images for: Molecular signature of COVID-19 prior to its exacerbation by multi-omics survey
Source: PLoS One. 2026 Jul 22;21(7):e0352423. doi: 10.1371/journal.pone.0352423 (PMC13390879; doi:10.1371/journal.pone.0352423)

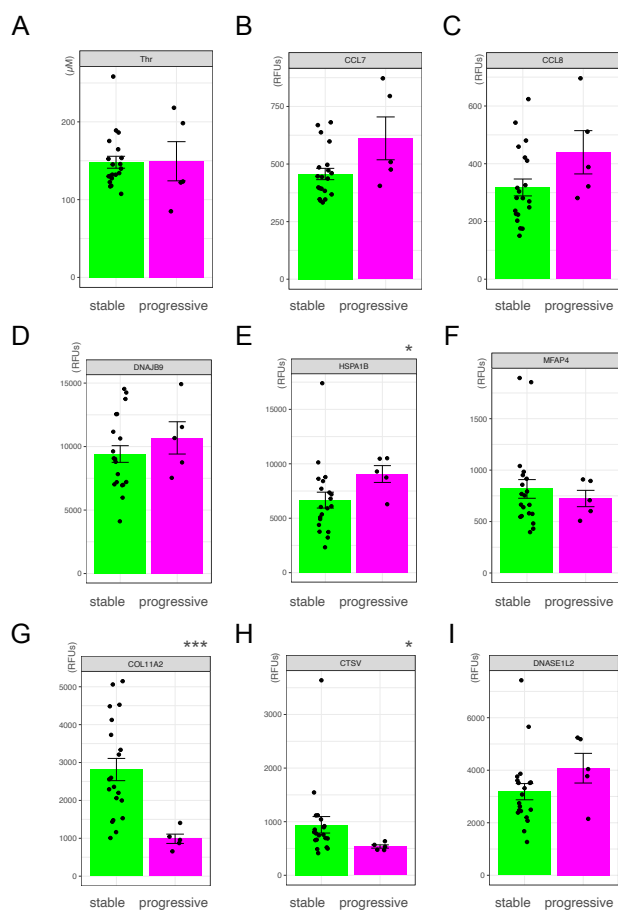

Fig S1. Molecular biomarker candidates after COVID-19 exacerbation

Supplement: S1 Fig — (A-I) Bar plots after admission using plasma samples (means ± SEM) (***p < 0.001, *p < 0.05). (PDF) [file pone.0352423.s001.pdf]

A

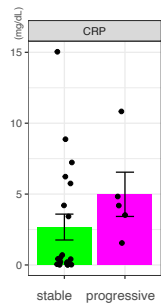

B

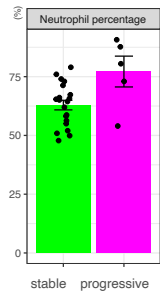

C

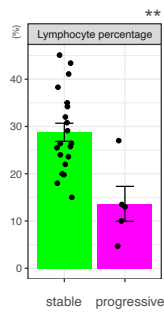

Fig S2. Clinical biomarker candidates after COVID-19 exacerbation

Supplement: S2 Fig — (A-C) Bar plots after admission using plasma samples (means ± SEM) (**p < 0.01). (PDF) [file pone.0352423.s002.pdf]
