# Supplementary material for: Molecular signature of COVID-19 prior to its exacerbation by multi-omics survey
Source: PLoS One. 2026 Jul 22;21(7):e0352423. doi: 10.1371/journal.pone.0352423 (PMC13390879; doi:10.1371/journal.pone.0352423)
Supplement: Attachment — Submitted filename: Response to Reviewers.docx [file pone.0352423.s011.docx]

The following is a point-by-point response to the reviewers’ comments, detailing the changes made to the manuscript.

**Reviewer #1**:

*Comment*:

- Tables 1 and 2 do not specify the sample size (n), nor do they appear in the results section. This value is only mentioned up to Figure 1 at the end of the document. Including this value in the table would be important for understanding the sample size, although very small samples are not usually representative and the statistical analysis may be affected (even though the analysis was performed with the entire patient population).

*Answer*:

We thank the reviewer for this helpful comment. As suggested, we have now included the sample size (n) in Tables 1 and 2 to clarify the number of patients analyzed. The tables have been revised accordingly.

(Results, page 12, table 1, page 15, table 2).

*Comment*:

- Unfortunately, the number of patients who received anti-inflammatory and immunomodulatory drugs is very small. It would have been interesting to evaluate whether the administration of these drugs influenced the expression of inflammatory molecules.

*Answer*:

We are grateful to the reviewer for this important comment. In our study, the primary analyses presented in Figures 2-5 were based on blood samples collected at the time of hospital admission, prior to the initiation of anti-inflammatory or immunomodulatory therapies and before the onset of severe symptoms. Therefore, these treatments are unlikely to have influenced the primary results. We agree with the reviewer that treatments administered after admission may have affected the longitudinal measurements presented in Supplementary Figures 1 and 2. However, we were unable to perform a meaningful analysis of treatment effects, as this aspect is not covered by the current study design. In addition, only a small number of patients received such therapies, and the types, timing, and dosages of medications varied substantially during the early pandemic period. We have added this point to the Discussion as a limitation of this study (Discussion, page 22, lines 529–533).

*Comment*:

- On the other hand, this study can lay the groundwork for monitoring these patients and evaluating whether those who expressed inflammatory molecules develop long COVID.

*Answer*:

We thank the reviewer for this insightful comment. We agree that this is an important direction for future research. However, long COVID–related clinical information was not systematically collected during the study period, and access to the medical records has already been closed. Therefore, we were unable to examine the relationship between inflammatory molecules and subsequent long COVID in the present study. We have added a statement in the Discussion section acknowledging this limitation and highlighting the need for future longitudinal studies (Discussion, page 22, lines 525–529).

**Reviewer #2**:

*Comment*:

1-If possible, it would be useful to add whether the patients were experiencing their first episode of COVID-19 (likely) or if they had received any vaccine (unlikely as Japan started vaccination on February 2021). This can help to contextualize some of the results and allow for future interpretations when a second infection of COVID-19 would look as likely as it would be observed now.

*Answer*:

We are grateful for the reviewer’s comment. We have added this information to the Discussion (Discussion, pages 19-20, lines 440–448). All patients included in this study experienced their first episode of COVID-19 infection. In addition, the study period preceded the introduction of COVID-19 vaccination in Japan in February 2021; therefore, none of the patients had received a COVID-19 vaccine. We believe this information helps clarify the clinical context of the cohort and facilitate interpretation of the results.

*Comment*:

2-In consideration of the lineages and changes in observed symptoms, it would be worth framing these results referring to the circulating lineages at that point. Consequently, any attempt to replicate such a study today will need to consider possible variations due to the virus lineage evolution.

*Answer*:

We thank the reviewer for this valuable suggestion. We have added a statement in the Discussion section noting that the samples were collected during 2020, when earlier SARS-CoV-2 lineages were circulating in Japan, and that the clinical manifestations observed in this study may reflect the SARS-CoV-2 lineages circulating during the study period. We also note that the continued evolution of SARS-CoV-2 may influence symptom profiles and should be considered when interpreting the findings or attempting to reproduce similar studies in the later phases of the pandemic (Discussion, pages 19, lines 436–440).

*Comment*:

3-Regarding the impaired interferon response, a factor to consider is the dysregulated activation of mast cells and the markers also predicting severe outcomes. Please take a look at: https://doi.org/10.3389/fimmu.2023.1166574

*Answer*:

We thank the reviewer for this insightful suggestion and for pointing out the relevant reference. We have revised the Discussion to include the potential contribution of the dysregulated mast cell activation to impaired interferon responses and severe COVID-19 outcomes, and we have cited the recommended article. We also note that histamine showed modest but statistically significant difference between the groups of our dataset, although it did not meet the predefined criteria for inclusion in the main marker selection analysis. We have briefly discussed this point in the revised manuscript (Discussion, pages 21-22, lines 494–510).

Sincerely,

Hideo Shindou

Department of Lipid Life Science, National Institute of Global Health and Medicine, Japan Institute for Health Security

Shinjuku-ku, Tokyo, 162-8655, Japan

Phone: +81-3-5273-5351

Email: shindou.h@jihs.go.jp
