# Supplementary material for: Molecular signature of COVID-19 prior to its exacerbation by multi-omics survey
Source: PLoS One. 2026 Jul 22;21(7):e0352423. doi: 10.1371/journal.pone.0352423 (PMC13390879; doi:10.1371/journal.pone.0352423)
Supplement: Peer review history

# Peer review: Decision Letter 0

**DOI:** 10.1371/journal.pone.0352423.r001

**Authors:** Jon Jacobs

3 Mar 2026

PONE-D-25-68902

Molecular signature of COVID-19 prior to its exacerbation by multi-omics survey

PLOS One

Dear Dr. Shindou,

Thank you for submitting your manuscript to PLOS ONE. After careful consideration, we feel that it has merit but does not fully meet PLOS ONE’s publication criteria as it currently stands. Therefore, we invite you to submit a revised version of the manuscript that addresses the points raised during the review process as noted by the reviewer's comments below.

Please submit your revised manuscript by Apr 17 2026 11:59PM. If you will need more time than this to complete your revisions, please reply to this message or contact the journal office at plosone@plos.org. When you're ready to submit your revision, log on to https://www.editorialmanager.com/pone/ and select the 'Submissions Needing Revision' folder to locate your manuscript file.

Please include the following items when submitting your revised manuscript:

- A letter that responds to each point raised by the academic editor and reviewer(s). You should upload this letter as a separate file labeled 'Response to Reviewers'.
- A marked-up copy of your manuscript that highlights changes made to the original version. You should upload this as a separate file labeled 'Revised Manuscript with Track Changes'.
- An unmarked version of your revised paper without tracked changes. You should upload this as a separate file labeled 'Manuscript'.

-->If you would like to make changes to your financial disclosure, please include your updated statement in your cover letter. Guidelines for resubmitting your figure files are available below the reviewer comments at the end of this letter.

If applicable, we recommend that you deposit your laboratory protocols in protocols.io to enhance the reproducibility of your results. Protocols.io assigns your protocol its own identifier (DOI) so that it can be cited independently in the future. For instructions see: https://journals.plos.org/plosone/s/submission-guidelines#loc-laboratory-protocols. Additionally, PLOS ONE offers an option for publishing peer-reviewed Lab Protocol articles, which describe protocols hosted on protocols.io. Read more information on sharing protocols at https://plos.org/protocols?utm_medium=editorial-email&utm_source=authorletters&utm_campaign=protocols.

We look forward to receiving your revised manuscript.

Kind regards,

Jon M. Jacobs, Ph.D.

Academic Editor

PLOS One

Journal Requirements:

When submitting your revision, we need you to address these additional requirements.

1. Please ensure that your manuscript meets PLOS ONE's style requirements, including those for file naming. The PLOS ONE style templates can be found at

https://journals.plos.org/plosone/s/file?id=wjVg/PLOSOne_formatting_sample_main_body.pdf and https://journals.plos.org/plosone/s/file?id=ba62/PLOSOne_formatting_sample_title_authors_affiliations.pdf

2. The ethical approval number(s) listed in the submission metadata does not match the approval number on the ethical approval document you provided. Please ensure that all approval numbers are correct.

3. Thank you for stating in your Funding Statement:

[This work was supported by AMED-CREST (grant number JP22gm0910011 to H. S.), the Japan Institute for Health Security (JIHS) Intramural Research Fund (22T001 to H. S., 24A1013 to H. S., and 24A2011 to H. S. and Y. Kita.). T. S. was supported by the AMED (grant number 20fk0108255 to T. S.) and Takeda Science Foundation.].

Please provide an amended statement that declares *all* the funding or sources of support (whether external or internal to your organization) received during this study, as detailed online in our guide for authors at http://journals.plos.org/plosone/s/submit-now.  Please also include the statement “There was no additional external funding received for this study.” in your updated Funding Statement.

Please include your amended Funding Statement within your cover letter. We will change the online submission form on your behalf.

4. When completing the data availability statement of the submission form, you indicated that you will make your data available on acceptance. We strongly recommend all authors decide on a data sharing plan before acceptance, as the process can be lengthy and hold up publication timelines. Please note that, though access restrictions are acceptable now, your entire data will need to be made freely accessible if your manuscript is accepted for publication. This policy applies to all data except where public deposition would breach compliance with the protocol approved by your research ethics board. If you are unable to adhere to our open data policy, please kindly revise your statement to explain your reasoning and we will seek the editor's input on an exemption. Please be assured that, once you have provided your new statement, the assessment of your exemption will not hold up the peer review process.

5. Thank you for stating the following in the Competing Interests section:

[The authors have declared that no competing interests exist.].

We note that one or more of the authors are employed by a commercial company: Lipidome Lab Co., Ltd.

1. Please provide an amended Funding Statement declaring this commercial affiliation, as well as a statement regarding the Role of Funders in your study. If the funding organization did not play a role in the study design, data collection and analysis, decision to publish, or preparation of the manuscript and only provided financial support in the form of authors' salaries and/or research materials, please review your statements relating to the author contributions, and ensure you have specifically and accurately indicated the role(s) that these authors had in your study. You can update author roles in the Author Contributions section of the online submission form.

Please also include the following statement within your amended Funding Statement.

“The funder provided support in the form of salaries for authors [insert relevant initials], but did not have any additional role in the study design, data collection and analysis, decision to publish, or preparation of the manuscript. The specific roles of these authors are articulated in the ‘author contributions’ section.”

If your commercial affiliation did play a role in your study, please state and explain this role within your updated Funding Statement.

2. Please also provide an updated Competing Interests Statement declaring this commercial affiliation along with any other relevant declarations relating to employment, consultancy, patents, products in development, or marketed products, etc.

Within your Competing Interests Statement, please confirm that this commercial affiliation does not alter your adherence to all PLOS ONE policies on sharing data and materials by including the following statement: "This does not alter our adherence to PLOS ONE policies on sharing data and materials.” (as detailed online in our guide for authors http://journals.plos.org/plosone/s/competing-interests) . If this adherence statement is not accurate and there are restrictions on sharing of data and/or materials, please state these. Please note that we cannot proceed with consideration of your article until this information has been declared.

Please include both an updated Funding Statement and Competing Interests Statement in your cover letter. We will change the online submission form on your behalf.

6. Please include your correct and complete ethics statement in the “Methods” section of your manuscript file, as indicated in the submission data. In your statement, please include the full name of the IRB or ethics committee who approved or waived your study, as well as whether or not you obtained informed written or verbal consent. If consent was waived for your study, please include this information in your statement as well.

7. We note that Figure 1 in your submission contains images which may be copyrighted. All PLOS content is published under the Creative Commons Attribution License (CC BY 4.0), which means that the manuscript, images, and Supporting Information files will be freely available online, and any third party is permitted to access, download, copy, distribute, and use these materials in any way, even commercially, with proper attribution. For more information, see our copyright guidelines: http://journals.plos.org/plosone/s/licenses-and-copyright.

We require you to either (1) present written permission from the copyright holder to publish these figures specifically under the CC BY 4.0 license, or (2) remove the figures from your submission:

1. You may seek permission from the original copyright holder of Figure 1 to publish the content specifically under the CC BY 4.0 license.

We recommend that you contact the original copyright holder with the Content Permission Form (http://journals.plos.org/plosone/s/file?id=7c09/content-permission-form.pdf) and the following text:

“I request permission for the open-access journal PLOS ONE to publish XXX under the Creative Commons Attribution License (CCAL) CC BY 4.0 (http://creativecommons.org/licenses/by/4.0/). Please be aware that this license allows unrestricted use and distribution, even commercially, by third parties. Please reply and provide explicit written permission to publish XXX under a CC BY license and complete the attached form.”

Please upload the completed Content Permission Form or other proof of granted permissions as an "Other" file with your submission.

In the figure caption of the copyrighted figure, please include the following text: “Reprinted from [ref] under a CC BY license, with permission from [name of publisher], original copyright [original copyright year].”

2. If you are unable to obtain permission from the original copyright holder to publish these figures under the CC BY 4.0 license or if the copyright holder’s requirements are incompatible with the CC BY 4.0 license, please either i) remove the figure or ii) supply a replacement figure that complies with the CC BY 4.0 license. Please check copyright information on all replacement figures and update the figure caption with source information. If applicable, please specify in the figure caption text when a figure is similar but not identical to the original image and is therefore for illustrative purposes only.

If the reviewer comments include a recommendation to cite specific previously published works, please review and evaluate these publications to determine whether they are relevant and should be cited. There is no requirement to cite these works unless the editor has indicated otherwise.

Please review your reference list to ensure that it is complete and correct. If you have cited papers that have been retracted, please include the rationale for doing so in the manuscript text, or remove these references and replace them with relevant current references. Any changes to the reference list should be mentioned in the rebuttal letter that accompanies your revised manuscript. If you need to cite a retracted article, indicate the article’s retracted status in the References list and also include a citation and full reference for the retraction notice.

[Note: HTML markup is below. Please do not edit.]

Reviewers' comments:

Reviewer's Responses to Questions

**Comments to the Author**

1. Is the manuscript technically sound, and do the data support the conclusions?

The manuscript must describe a technically sound piece of scientific research with data that supports the conclusions. Experiments must have been conducted rigorously, with appropriate controls, replication, and sample sizes. The conclusions must be drawn appropriately based on the data presented.

Reviewer #1: Yes

Reviewer #2: Yes

**********

2. Has the statistical analysis been performed appropriately and rigorously?

Reviewer #1: Yes

Reviewer #2: Yes

**********

3. Have the authors made all data underlying the findings in their manuscript fully available?

The PLOS Data policy requires authors to make all data underlying the findings described in their manuscript fully available without restriction, with rare exception (please refer to the Data Availability Statement in the manuscript PDF file). The data should be provided as part of the manuscript or its supporting information, or deposited to a public repository. For example, in addition to summary statistics, the data points behind means, medians and variance measures should be available. If there are restrictions on publicly sharing data—e.g. participant privacy or use of data from a third party—those must be specified.

Reviewer #1: Yes

Reviewer #2: Yes

**********

4. Is the manuscript presented in an intelligible fashion and written in standard English?

PLOS ONE does not copyedit accepted manuscripts, so the language in submitted articles must be clear, correct, and unambiguous. Any typographical or grammatical errors should be corrected at revision, so please note any specific errors here.

Reviewer #1: Yes

Reviewer #2: Yes

**********

5. Review Comments to the Author

Please use the space provided to explain your answers to the questions above. You may also include additional comments for the author, including concerns about dual publication, research ethics, or publication ethics. (Please upload your review as an attachment if it exceeds 20,000 characters)

Reviewer #1: Following peer review of this document, we found it to be a highly relevant study from an omics perspective.

- Tables 1 and 2 do not specify the sample size (n), nor do they appear in the results section. This value is only mentioned up to Figure 1 at the end of the document. Including this value in the table would be important for understanding the sample size, although very small samples are not usually representative and the statistical analysis may be affected (even though the analysis was performed with the entire patient population).

- Unfortunately, the number of patients who received anti-inflammatory and immunomodulatory drugs is very small. It would have been interesting to evaluate whether the administration of these drugs influenced the expression of inflammatory molecules.

- On the other hand, this study can lay the groundwork for monitoring these patients and evaluating whether those who expressed inflammatory molecules develop long COVID.

I believe that by modifying the document with the minor corrections suggested, it could be considered for publication.

Reviewer #2: Shindou et al. present a multi-omics analysis searching for markers that can predict the clinical progression of COVID-19 cases. The addition of lipidomics and metabolomics allows wider interpretation of the observed clinical signs. The study is well designed and properly analyzed, despite the limited number of samples. I have some minor suggestions for the authors to consider:

1-If possible, it would be useful to add whether the patients were experiencing their first episode of COVID-19 (likely) or if they had received any vaccine (unlikely as Japan started vaccination on February 2021). This can help to contextualize some of the results and allow for future interpretations when a second infection of COVID-19 would look as likely as it would be observed now.

2-In consideration of the lineages and changes in observed symptoms, it would be worth framing these results referring to the circulating lineages at that point. Consequently, any attempt to replicate such a study today will need to consider possible variations due to the virus lineage evolution.

3-Regarding the impaired interferon response, a factor to consider is the dysregulated activation of mast cells and the markers also predicting severe outcomes. Please take a look at: https://doi.org/10.3389/fimmu.2023.1166574

**********

6. PLOS authors have the option to publish the peer review history of their article (what does this mean?). If published, this will include your full peer review and any attached files.

If you choose “no”, your identity will remain anonymous but your review may still be made public.

**Do you want your identity to be public for this peer review?**  For information about this choice, including consent withdrawal, please see our Privacy Policy.

Reviewer #1: **Yes:**  Luis Del Carpio-Orantes

Reviewer #2: No

**********

[NOTE: If reviewer comments were submitted as an attachment file, they will be attached to this email and accessible via the submission site. Please log into your account, locate the manuscript record, and check for the action link "View Attachments". If this link does not appear, there are no attachment files.]

To ensure your figures meet our technical requirements, please review our figure guidelines: https://journals.plos.org/plosone/s/figures

You may also use PLOS’s free figure tool, NAAS, to help you prepare publication quality figures: https://journals.plos.org/plosone/s/figures#loc-tools-for-figure-preparation.

NAAS will assess whether your figures meet our technical requirements by comparing each figure against our figure specifications.

# Author comment: Author response to Decision Letter 1

**DOI:** 10.1371/journal.pone.0352423.r002

2 Jun 2026

The following is a point-by-point response to the reviewers’ comments, detailing the changes made to the manuscript.

Reviewer #1:

Comment:

- Tables 1 and 2 do not specify the sample size (n), nor do they appear in the results section. This value is only mentioned up to Figure 1 at the end of the document. Including this value in the table would be important for understanding the sample size, although very small samples are not usually representative and the statistical analysis may be affected (even though the analysis was performed with the entire patient population).

Answer:

We thank the reviewer for this helpful comment. As suggested, we have now included the sample size (n) in Tables 1 and 2 to clarify the number of patients analyzed. The tables have been revised accordingly.

(Results, page 12, table 1, page 15, table 2).

Comment:

- Unfortunately, the number of patients who received anti-inflammatory and immunomodulatory drugs is very small. It would have been interesting to evaluate whether the administration of these drugs influenced the expression of inflammatory molecules.

Answer:

We are grateful to the reviewer for this important comment. In our study, the primary analyses presented in Figures 2-5 were based on blood samples collected at the time of hospital admission, prior to the initiation of anti-inflammatory or immunomodulatory therapies and before the onset of severe symptoms. Therefore, these treatments are unlikely to have influenced the primary results. We agree with the reviewer that treatments administered after admission may have affected the longitudinal measurements presented in Supplementary Figures 1 and 2. However, we were unable to perform a meaningful analysis of treatment effects, as this aspect is not covered by the current study design. In addition, only a small number of patients received such therapies, and the types, timing, and dosages of medications varied substantially during the early pandemic period. We have added this point to the Discussion as a limitation of this study (Discussion, page 22, lines 529–533).

Comment:

- On the other hand, this study can lay the groundwork for monitoring these patients and evaluating whether those who expressed inflammatory molecules develop long COVID.

Answer:

We thank the reviewer for this insightful comment. We agree that this is an important direction for future research. However, long COVID–related clinical information was not systematically collected during the study period, and access to the medical records has already been closed. Therefore, we were unable to examine the relationship between inflammatory molecules and subsequent long COVID in the present study. We have added a statement in the Discussion section acknowledging this limitation and highlighting the need for future longitudinal studies (Discussion, page 22, lines 525–529).

Reviewer #2:

Comment:

1-If possible, it would be useful to add whether the patients were experiencing their first episode of COVID-19 (likely) or if they had received any vaccine (unlikely as Japan started vaccination on February 2021). This can help to contextualize some of the results and allow for future interpretations when a second infection of COVID-19 would look as likely as it would be observed now.

Answer:

We are grateful for the reviewer’s comment. We have added this information to the Discussion (Discussion, pages 19-20, lines 440–448). All patients included in this study experienced their first episode of COVID-19 infection. In addition, the study period preceded the introduction of COVID-19 vaccination in Japan in February 2021; therefore, none of the patients had received a COVID-19 vaccine. We believe this information helps clarify the clinical context of the cohort and facilitate interpretation of the results.

Comment:

2-In consideration of the lineages and changes in observed symptoms, it would be worth framing these results referring to the circulating lineages at that point. Consequently, any attempt to replicate such a study today will need to consider possible variations due to the virus lineage evolution.

Answer:

We thank the reviewer for this valuable suggestion. We have added a statement in the Discussion section noting that the samples were collected during 2020, when earlier SARS-CoV-2 lineages were circulating in Japan, and that the clinical manifestations observed in this study may reflect the SARS-CoV-2 lineages circulating during the study period. We also note that the continued evolution of SARS-CoV-2 may influence symptom profiles and should be considered when interpreting the findings or attempting to reproduce similar studies in the later phases of the pandemic (Discussion, pages 19, lines 436–440).

Comment:

3-Regarding the impaired interferon response, a factor to consider is the dysregulated activation of mast cells and the markers also predicting severe outcomes. Please take a look at: https://doi.org/10.3389/fimmu.2023.1166574

Answer:

We thank the reviewer for this insightful suggestion and for pointing out the relevant reference. We have revised the Discussion to include the potential contribution of the dysregulated mast cell activation to impaired interferon responses and severe COVID-19 outcomes, and we have cited the recommended article. We also note that histamine showed modest but statistically significant difference between the groups of our dataset, although it did not meet the predefined criteria for inclusion in the main marker selection analysis. We have briefly discussed this point in the revised manuscript (Discussion, pages 21-22, lines 494–510).

Sincerely,

Hideo Shindou

Department of Lipid Life Science, National Institute of Global Health and Medicine, Japan Institute for Health Security

Shinjuku-ku, Tokyo, 162-8655, Japan

Phone: +81-3-5273-5351

Email: shindou.h@jihs.go.jp

Attachment Submitted filename: Response to Reviewers.docx

# Editor report: Decision Letter 1

**DOI:** 10.1371/journal.pone.0352423.r003

**Authors:** Jon Jacobs, Jon Jacobs

11 Jun 2026

Molecular signature of COVID-19 prior to its exacerbation by multi-omics survey

PONE-D-25-68902R1

Dear Dr. Shindou,

We’re pleased to inform you that your manuscript has been judged scientifically suitable for publication and will be formally accepted for publication once it meets all outstanding technical requirements.

Within one week, you’ll receive an e-mail detailing the required amendments. When these have been addressed, you’ll receive a formal acceptance letter and your manuscript will be scheduled for publication.

An invoice will be generated when your article is formally accepted. Please note, if your institution has a publishing partnership with PLOS and your article meets the relevant criteria, all or part of your publication costs will be covered. Please make sure your user information is up-to-date by logging into Editorial Manager at Editorial Manager® and clicking the ‘Update My Information' link at the top of the page. For questions related to billing, please contact billing support.

If your institution or institutions have a press office, please notify them about your upcoming paper to help maximize its impact. If they’ll be preparing press materials, please inform our press team as soon as possible -- no later than 48 hours after receiving the formal acceptance. Your manuscript will remain under strict press embargo until 2 pm Eastern Time on the date of publication. For more information, please contact onepress@plos.org.

Kind regards,

Jon M. Jacobs, Ph.D.

Academic Editor

PLOS One

Additional Editor Comments (optional):

Reviewers' comments:

# Editor report: Acceptance letter

**DOI:** 10.1371/journal.pone.0352423.r004

**Authors:** Jon Jacobs, Jon Jacobs

PONE-D-25-68902R1

PLOS One

Dear Dr. Shindou,

I'm pleased to inform you that your manuscript has been deemed suitable for publication in PLOS One. Congratulations! Your manuscript is now being handed over to our production team.

At this stage, our production department will prepare your paper for publication. This includes ensuring the following:

* All references, tables, and figures are properly cited

* All relevant supporting information is included in the manuscript submission,

* There are no issues that prevent the paper from being properly typeset

You will receive further instructions from the production team, including instructions on how to review your proof when it is ready. Please keep in mind that we are working through a large volume of accepted articles, so please give us a few days to review your paper and let you know the next and final steps.

Lastly, if your institution or institutions have a press office, please let them know about your upcoming paper now to help maximize its impact. If they'll be preparing press materials, please inform our press team within the next 48 hours. Your manuscript will remain under strict press embargo until 2 pm Eastern Time on the date of publication. For more information, please contact onepress@plos.org.

You will receive an invoice from PLOS for your publication fee after your manuscript has reached the completed accept phase. If you receive an email requesting payment before acceptance or for any other service, this may be a phishing scheme. Learn how to identify phishing emails and protect your accounts at https://explore.plos.org/phishing.

If we can help with anything else, please email us at customercare@plos.org.

Thank you for submitting your work to PLOS ONE and supporting open access.

Kind regards,

PLOS ONE Editorial Office Staff

on behalf of

Dr Jon M. Jacobs

Academic Editor

PLOS One
